# Conservation of mRNA operon formation in control of the heat shock response in mammalian cells

**DOI:** 10.1126/sciadv.adu0315

**Published:** 2025-12-05

**Authors:** Emese Pataki, Jeffrey E. Gerst

**Affiliations:** Department of Molecular Genetics, Weizmann Institute of Science, Rehovot 7610001, Israel.

## Abstract

Prokaryotes use polycistronic transcription (operons) to express multiple messenger RNAs (mRNAs) from a single promoter to coexpress functionally related genes. However, how do eukaryotes, which express monocistronic messages, achieve the same regulation? Previously, we demonstrated that yeast uses RNA operons, i.e., mRNAs assembled in trans (transperons), to control multiple cellular pathways such as the heat shock response (HSR). As the HSR is conserved from yeast to mammals, we used single-molecule RNA labeling and pulldown techniques to demonstrate that mammalian heat shock protein (HSP) mRNAs also form operons upon transcription during heat stress. HSP RNA operon formation is dependent on the heat shock factor 1 transcription factor and intra- and interchromosomal interactions between the HSP genes. Work in yeast identified a conserved RNA sequence motif and histone H4 functions that act downstream thereof to regulate transperon assembly. Our work highlights the evolutionarily conserved regulation of the HSR and for RNA operons in eukaryotic gene regulation.

## Teaser

Eukaryotes coordinate their response to heat shock through an evolutionarily conserved mechanism involving mRNA operon expression.

## INTRODUCTION

Prokaryotic organisms efficiently regulate gene expression through polycistronic transcription units, such as operons, allowing for the simultaneous expression of multiple genes from a single promoter in response to environmental stimuli (*1*). While eukaryotes lack DNA-based operons, it has been suggested that they may have evolved analogous mechanisms to coordinate gene expression (i.e., RNA operons) (*2*, *3*). Recent studies have shown that eukaryotic cells use various genome organization strategies to regulate gene expression, including chromatin looping, inducible transcriptional condensates, and the formation of “transcriptional factories” (*4*, *5*). These mechanisms allow for the clustering of genes involved in shared biological pathways, enhancing the efficiency and coordination of gene expression in response to stimuli (*3*, *6*–*8*). For example, in yeast, transcriptional condensates, like the super-enhancers found in mammalian cells, enable the rapid activation of stress-responsive genes (*4*, *6*). In yeast, Chowdhary *et al.* demonstrated that heat shock factor 1 (HSF1) forms transcriptional condensates with the RNA polymerase II (Pol II) and Mediator complexes to mediate intergenic interactions between heat shock protein (HSP) genes (*6*). This interaction results in coordinated gene expression and transcriptional control across different chromosomes. Work by our laboratory extended these findings by identifying the existence of transperons, multiplexed assemblies of cotranscribed mRNAs bound in trans that correlate with the presence of both intra- and interchromosomal interactions and encode proteins that function on the same cellular pathway(s) (*3*, *9*). Consistent with the hypothesis of eukaryotic RNA operons, we identified transperons encoding components of the mating pathway of either yeast haplotype, another for mitochondrial outer membrane proteins, and one composed of HSP transcripts (e.g., *HSP104*, *HSP12*, *HSP82*, *SSA2*, and *SSA4*; HSP transperon) that was found to be crucial for the yeast heat shock response (HSR) (*9*). Notably, transperon assembly and function depends upon histone H4, and its depletion leads to defects in RNA multiplexing and loss of physiological functions (*9*). Transperon formation also appears regulated by histone H4 acetylation, which may suggest a role at the level of transcription (*3*).

From yeast to humans, the same core group of chaperone-encoding genes is induced by heat shock and under the regulation of the HSF1 transcription factor, the key regulator of HSR (*10*–*12*). Therefore, the HSR is an excellent system to study eukaryotic gene regulation based on its robust induction and conserved components. Despite its name, the HSR is not only sensitive to temperature change, but is also activated by oxidative stress, glucose deprivation, and the presence of misfolded proteins. HSF1 is a winged helix DNA binding heat shock transcription factor that recognizes a motif (i.e., heat shock response element; HSE) found in the promoter of chaperone genes (*12*–*14*). Under basal cellular conditions in mammalian cells, inactive HSF1 resides in the cytoplasm forming a complex together with HSP40, HSP70, HSP90, and the T-complex protein (TCP1) ring complex (*10*, *11*). Under stress conditions, HSF1 dissociates from the complex, trimerizes, and translocates to the nucleus where it binds its target genes to initiate transcriptional activation of chaperones, including HSP90, HSP70, and small HSPs, which help to refold any denatured proteins and maintain cellular proteostasis. After protein homeostasis has been restored, the excess of HSP70 acts as a negative feedback signal upon HSF1, which facilitates its relocation to the cytosol (*15*–*17*). Given that the HSR is highly conserved and that both yeast and mammalian HSP genes undergo coalescence upon heat shock (*8*, *9*), we hypothesized that HSF1-mediated transcription in mammalian cells might also lead to HSP transperon formation to facilitate a coordinated cellular response to heat stress and proteotoxic conditions. Using single-molecule fluorescence in situ hybridization (smFISH), chromatin conformation capture (3C), and single-species RNA pulldown techniques, we report HSP transperon formation in mouse embryo fibroblasts (MEFs) exposed to brief heat shock. Mammalian HSP transperon formation is dependent upon the exposure to heat shock and necessitates the presence of HSF1, as both HSP gene interactions and transperon formation were greatly reduced in cells lacking HSF1. Thus, mRNA operon/transperon formation appears conserved in eukaryotes at least with respect to the HSR. Given the complexity of mammalian systems, transperon study may offer important insights into gene regulation and open avenues for therapeutic interventions targeting stress response pathways.

## RESULTS

### Inter- and intrachromosomal association between mammalian heat shock genes

**Fig. 1.**
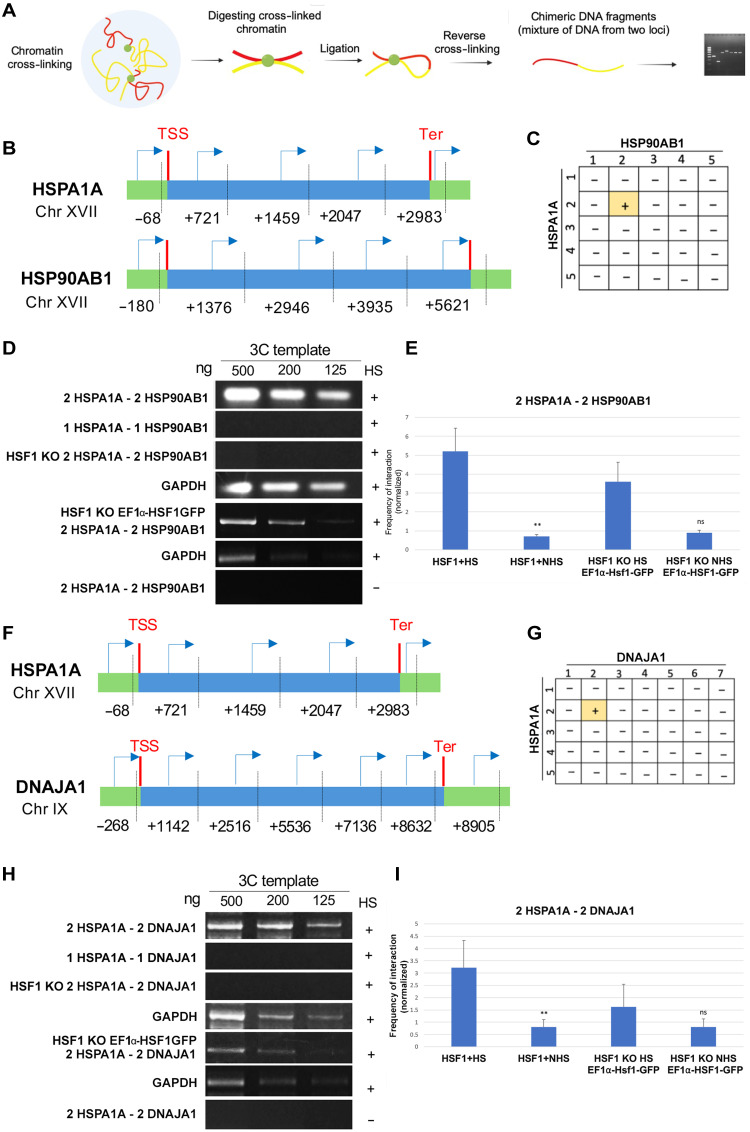
Intergenic interactions between mammalian HSP genes. (**A**) Schematic representation of the 3C technique used to assess physical interactions between gene loci. (**B** and **F**) Gene maps of HSPA1A and HSP90AB1 (B), and HSPA1A and DNAJA1 (F), showing locations of the Taq I restriction sites (vertical dashed lines) and the primers used for 3C-PCR (arrows). Site numbering is relative to the ATG start codon (+1). Forward primers were identical to the sense strand and positioned proximal to the corresponding Taq I sites. Untranslated regions and open reading frames are color-coded as indicated; transcription start (TSS) and termination (Ter) sites are labeled. (**C** and **G**) Summary matrices indicating whether interactions were detected between the gene loci using 3C-PCR with the indicated primer pairs. “+” denotes successful amplification (i.e., an interaction); “–” indicates no detectable product (i.e., no interaction). (**D** and **H**) Agarose gel electrophoresis of the PCR products amplified from 3C-processed DNA using primer pairs shown in (B) and (F). Lanes show PCR reactions with varying template DNA input (nanograms). Glyceraldehyde-3-phosphate dehydrogenase (GAPDH) DNA was used as a positive PCR control. PCR products were electrophoresed on 1% agarose gels and stained with ethidium bromide. (**E** and **I**) Quantification of interaction frequencies as determined by locus-specific qPCR, as described in Materials and Methods. **P < 0.01; ns, not significant. Error bars represent the SD. HS, heat shock; NHS, no heat shock.

Previous work has demonstrated that intergenic associations occur between yeast HSP genes under heat stress leading to transperon formation and the HSR. To investigate whether mammalian HSP genes also exhibit gene coalescence during heat shock, we used the 3C technique of Chowdhary *et al.* (*18*). Using DNA samples from MEFs exposed to heat shock (42°C, 1 hour) and polymerase chain reaction (PCR), we identified two interactions: an intrachromosomal interaction between HSPA1A (HSP70) and HSP90AB1 (HSP90) (Fig. 1, B to E), and an interchromosomal interaction between HSPA1A and DNAJA1 (HSP40) (Fig. 1, F to I). In both cases, the 5′ coding regions of HSPA1A were involved, suggesting a hotspot for interactions within this region as previously seen in yeast (*6*, *18*). Interactions were confirmed by both PCR (Fig. 1, D and H) and DNA quantitative PCR (qPCR) (Fig. 1, E and I) and were found to be specific, as no interactions were observed between other regions of the genes. Notably, the knockout of HSF1 (KO; HSF1^−/−^) abolished interactions between HSPA1A and either of the HSP90AB1 and DNAJA1 genes (Fig. 1, D and H, respectively). However, stably reintroducing HSF1–green fluorescent protein (GFP) by mild expression under the elongation factor 1 alpha (EF1α) promoter restored these interactions (Fig. 1, D and H). Comparable levels of HSF1 expression were observed in both wild-type (WT) MEFs and HSF1 KO cells expressing HSF1 from the EF1α promoter (fig. S1A). Therefore, we conclude that mouse HSP genes can physically interact and that this gene coalescence is HSF1-dependent.

Despite the knowledge that HSP70, HSP90, and HSP40 are commonly regulated by HSF1, we did not observe an intergenic interaction between HSP90AB1 and DNAJA1 (fig. S1B). This discrepancy, however, might be due to the existence of multiple HSP isoforms within each HSP family in mammalian cells. Based on transcriptome sequencing, we previously found that there are four different DNAJA isoforms (e.g., DNAJA1, DNAJA2, DNAJA3, and DNAJA4) and >30 different HSP isoforms transcribed in MEFs under non–heat shock conditions, with varying degrees of expression (data file S1) (*19*).

### HSP mRNAs colocalize in MEF cells upon exposure to heat shock

**Fig. 2.**
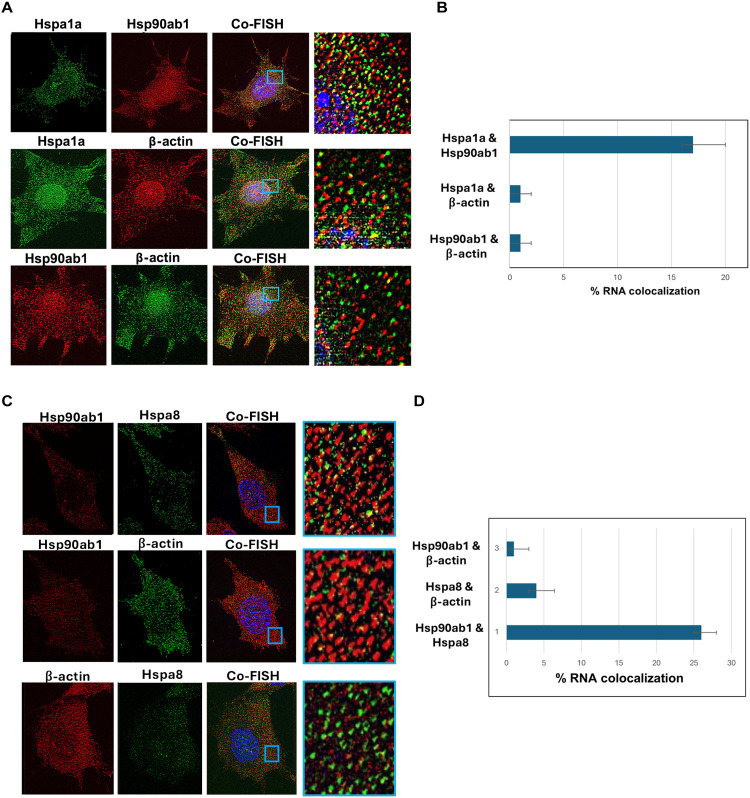
HSP mRNAs colocalize upon heat shock. (**A** and **C**) Representative smFISH images of MEFs that underwent heat shock (HS) or did not (NHS). The cells were processed for smFISH labeling using sequence-specific FISH probes complementary to HSPA1A, HSP90AB1, and HSPA8 mRNAs, prior to labeling with 4′,6-diamidino-2-phenylindole (blue). HSPA1A and HSPA8 mRNAs were labeled with Quasar 670–labeled oligonucleotides (green channel), whereas HSP90AB1 mRNA was labeled with Quasar 570–labeled oligonucleotides (red channel). Images were filtered and analyzed using FISH-quant. Co-FISH indicates the merge of all three signals. The blue squares in the co-FISH panels indicate a zoomed-in image from the merged signals. (**B** and **D**) Histograms of the data obtained from three biological replicates. Error bars represent the SD.

Because HSPA1A and HSP90AB1 physically interact on the same chromosome and undergo gene coalescence, we investigated whether these genes also interact at the RNA level. To address this, we performed smFISH using specific fluorescent oligonucleotide probes (Fig. 2 and data file S1). First, to assess HSP mRNA expression levels in MEFs, we extracted RNA from cells maintained at 37°C or exposed to heat shock (42°C, 1 hour). As shown in fig. S2, several HSP mRNAs were expressed under normal growth conditions, but their expression levels strongly increased following heat stress. In contrast, HSP mRNA levels were greatly reduced after heat shock in HSF1^−/−^ KO cells, with minimal expression observed under normal growth conditions (fig. S2).

We used smFISH to examine for mRNA interactions, targeting HSPA1A mRNA with sequence-specific probes labeled with Quasar 670 and HSP90AB1 mRNA with probes labeled with Quasar 570 (Fig. 2A). The number of mRNA signals was quantified using FISH-quant (Fig. 2B) (*20*). No HSPA1A mRNA signals were observed in cells not exposed to heat shock, while HSP90AB1 mRNA spots were observed (fig. S3A). In contrast, both HSPA1A mRNA and HSP90AB1 mRNA signals were detected upon heat shock (Fig. 2A), and quantification revealed that 17% of HSPA1A mRNAs colocalized with HSP90AB1 (Fig. 2B). Because HSPA1A mRNA is heat shock–induced, colocalization might be expected to occur postinduction and therefore only partially overlap with constitutively expressed HSP90AB1 mRNA. As a control, we examined for RNA colocalization between β-actin mRNA and either HSPA1A or HSP90AB1 but did not observe any interaction.

As both HSPA1A and HSP90AB1 reside on Chr XVII, we examined whether HSP90AB1 mRNA could colocalize with another HSP mRNA when the genes are located on two different chromosomes (e.g., HSP90AB1 on Chr XVII and HSPA8 on Chr IX). Representative smFISH images (Fig. 2C) show the labeled HSP90AB1 and HSPA8 mRNAs and their level of colocalization (Fig. 2D). The results show that 26% of HSPA8 mRNAs colocalized with HSP90AB1 mRNAs upon heat shock. Again, little interaction was observed between either HSP90AB1 or HSPA8 mRNA and β-actin mRNA. Thus, the HSP mRNA interactions observed appear specific.

We also observed colocalization of the HSPA1A and HSP90AB1 as well as HSPA8 and HSP90AB1 transcription sites under heat shock conditions, providing further evidence for coordinated regulation of these genes (fig. S3, B and C). Moreover, our laboratory has demonstrated that full-length mRNA molecules can transfer between mammalian cells through thin, open-ended cellular protrusions known as tunneling nanotubes (TNTs) (*19*, *21*, *22*). Specifically, we observed that the HSP90AB1 and HSPA1A mRNAs may colocalize within TNTs (see representative image; fig. S3D), suggesting that transperons can undergo transport from one cell to another via TNTs.

We also examined whether the addition of translation inhibitors, such as puromycin or cycloheximide, might affect transperon formation and thereby HSP RNA colocalization. Cells were subjected to heat shock at 42°C for 1 hour, followed by recovery for 1 hour at 37°C. Within the last 30 min of the recovery, cells were treated with the inhibitors; however, no effect upon HSP90AB1 and HSPA1A mRNA colocalization was observed (fig. S3E).

### mRNAs encoding mammalian HSPs multiplex to form a heat shock–enhanced transperon

**Fig. 3.**
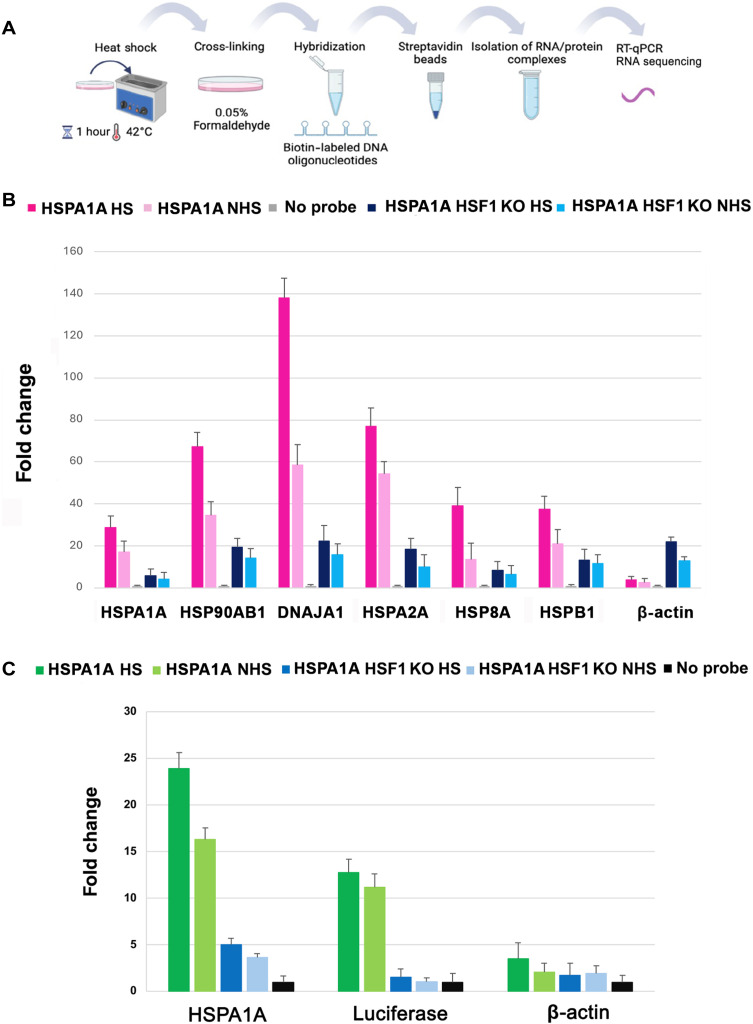
HSF1 and the HSP promoter containing a HSE drive HSP RNA multiplexing. (**A**) Schematic of the RNA pulldown technique using biotin-labeled DNA oligonucleotides. (**B**) WT MEF and HSF1^−/−^ KO MEFs were either exposed to heat shock (1 hour, 42°C; HS) or maintained at 37°C (NHS) prior to fixation and the RNA pulldown procedure. RT-qPCR was performed using gene-specific oligonucleotides. Pulldown RNA levels were normalized to transcript levels in the total cell lysate (TCL) and are presented relative to that of the “no probe” control, which represents background signal in the absence of added biotinylated oligonucleotides (negative control). Data represent the mean of three biological replicates, with error bars indicating SD. (**C**) 6xHSE-RLuc plasmid was transiently expressed in WT and HSF1^−/−^ KO MEF cells and either subjected to heat shock at 42°C for 1 hour or maintained at 37°C prior to fixation and pulldown procedure. RT-qPCR was performed using gene-specific oligonucleotides. Pulldown RNA levels were normalized to transcript levels in the TCL and are presented relative to the “no probe” control, which represents background signal in the absence of biotinylated oligonucleotides. Data represent the mean of three biological replicates.

To investigate whether mRNAs encoding mammalian HSPs multiplex to form transperons, we used an RNA-specific pulldown assay created by Torres *et al.* (*23*) with some modifications, as detailed in Materials and Methods. We designed biotin-labeled DNA oligonucleotides complementary to HSPA1A and hybridized them with whole cell extracts derived from formaldehyde–cross-linked WT and HSF1 KO cells that were incubated at either 42°C to induce heat shock or maintained at 37°C as a control. Next, we used immobilized streptavidin to precipitate the HSPA1A RNA followed by reverse transcription PCR (RT-PCR) to detect coprecipitated mRNAs after normalization for expression (Fig. 3A).

We found that HSP mRNAs previously shown to undergo intergenic interactions and mRNA colocalization, such as HSP90AB1, DNAJA1, and HSPA8 (Figs. 1 and 2), along with HSPB1, showed coprecipitation with HSPA1A mRNA from WT cells that was more robust upon the exposure of cells to heat shock (Fig. 3B). The different RNAs precipitated displayed varying levels of expression within the multiplex, which may reflect the stoichiometry of the RNA complex.

Furthermore, HSF1 was once again identified as a critical regulator of RNA multiplexing, as in its absence (i.e., HSF KO cells), the amount of HSP mRNAs that coprecipitated with HSPA1A RNA was greatly reduced. These results demonstrate that the HSP RNA transperon is conserved in evolution.

Because HSF1 plays an essential role in promoting formation of the heat shock transperon and the HSR in both yeast and mammalian cells, we hypothesized that the presence of HSEs in a given gene promoter might be sufficient to confer gene coalescence, cotranscription, and assembly into the RNA operon upon heat shock. This is because active oligomeric HSF1 has been demonstrated to undergo liquid-liquid phase separation on the promoters of HSP genes to form a condensate that allows for efficient Pol II transcription (*6*). To test the possibility that the HSE is necessary and sufficient to confer RNA incorporation into the HSP transperon, we transfected MEFs with plasmids either expressing pGLuc or 6xHSE-Rluc, the latter containing six copies of the HSE, into both WT and HSF1 KO cells and precipitated HSPA1A RNA. Upon RT-PCR analysis, we found that HSE-luciferase RNA was incorporated into the RNA multiplex in a heat shock– and HSF1-dependent manner (Fig. 3C). In contrast, β-actin mRNA was not incorporated under any condition. As expected, the GLuc luciferase gene lacking the HSEs did not appear in the multiplex (fig. S4). Thus, HSEs appear necessary and sufficient to confer mRNA participation in multiplexing, indicating an essential role for this transcriptional element in the control of transperon formation.

### A conserved sequence motif within the coding region of HSP mRNAs promotes RNA multiplexing

**Fig. 4.**
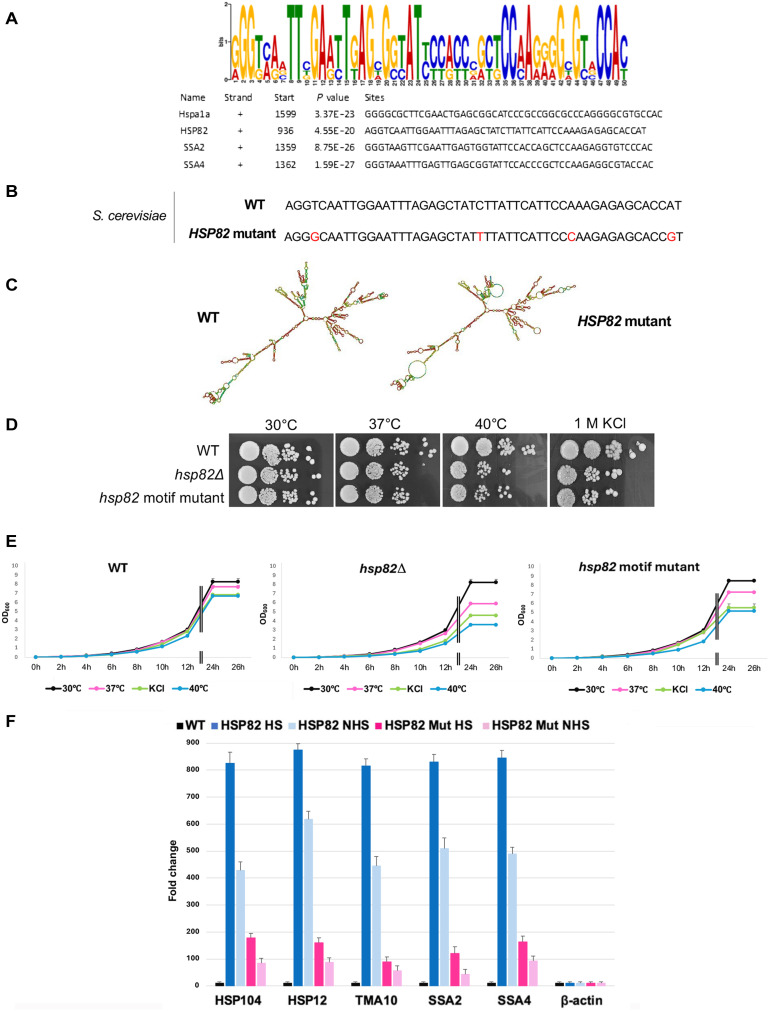
A conserved RNA motif facilitates HSP transperon assembly and function in yeast. (**A**) A motif in the coding region is common to the *M. musculus* HSPA1A and the *S. cerevisiae HSP82*, *SSA2*, and *SSA4* HSP genes. MEME-ChIP analysis of all four HSP mRNAs revealed a consensus motif, shown schematically as a sequence logo based on nucleotide representation. (**B**) The *S. cerevisiae HSP82* (WT) and motif mutant with alterations that change conserved nucleotides without affecting the protein sequence are shown. (**C**) The *HSP82* mutant has an altered secondary structure. RNA-fold was used to model the native and mutant versions of *HSP82* mRNA. (**D**) Mutation of the consensus motif in *HSP82* leads to partial loss of function under high temperature and osmotic stress. Serial dilutions of cells bearing *HSP82* or the *HSP82* motif mutant, as well as *hsp82*Δ cells were spotted onto yeast extract, peptone, and dextrose (YPD) medium with or without 1 M KCl and incubated for 48 hours at the indicated temperatures. (**E**) Quantitative growth assays of cells bearing *HSP82*, the *HSP82* motif mutant, or *hsp82*Δ were performed in YPD with/without 1 M KCl, for up to 26 hours at indicated temperatures. Growth was monitored at OD_600_, with averages of three technical replicates plotted. Error bars (SD) are shown; those less than 0.1 SD are not visible. (**F**) Mutation of the consensus motif in *HSP82* leads to a decrease in HSP mRNA multiplexing. Cells expressing MS2 aptamer-tagged *HSP82* or *HSP82* motif mutant (*HSP82 Mut*) were exposed to heat shock (10 min, 40°C; HS) or maintained at 30°C (NHS) prior to RaPID mRNA pulldown. qPCR was performed using gene-specific oligonucleotides, and pulldown RNA levels were normalized to the TCL and shown relative to samples lacking the MS2 tag, which serve as a control. Data represent the mean of three biological replicates with error bars indicating SD. h, hours.

Because the HSR genes are conserved across species from yeast to humans, we performed a bioinformatics analysis to identify recognizable motifs in their sequences (*24*). Using MEME-ChIP analysis (https://nbcr.net/meme/meme/cgi-bin/meme-chip.cgi), we identified a conserved motif within the coding regions of the mammalian HSP70 (HSPA1A) and yeast HSP70 (*SSA2* and *SSA4*) and HSP90 (*HSP82*) orthologs in *Mus musculus* and *Saccharomyces cerevisiae*, respectively, represented by a sequence logo based on nucleotide composition (Fig. 4A). To determine whether this motif contributes to RNA multiplexing, we generated a mutated version of the yeast *HSP82* gene by substituting four conserved nucleotides within the motif with synonymous mutations that did not alter the amino acid sequence (*hsp82^mut^*; Fig. 4B). Structural analysis using RNA-fold revealed that *hsp82^mut^* was distorted as compared to the native *HSP82* sequence (Fig. 4C). These mutations did not affect the expression level of *HSP82* (fig. S5A). In addition, we examined the phenotype of the *hsp82^mut^* strain under temperature and osmotic stress conditions and found that it was intermediate between the WT cells and the *hsp82*Δ deletion mutant (Fig. 4, D and E), indicating that these mutations resulted in a partial loss of function.

Given the effect upon cell physiology, we examined HSP transperon formation in *hsp82^mut^* cells. We used RaPID, an RNA pulldown technique developed in our laboratory that uses the MS2 coat protein fused to GFP and streptavidin-binding peptide to precipitate MS2 aptamer-tagged mRNAs (*9*, *25*, *26*) from cells bearing the *HSP82* and *hsp82^mut^* genes tagged at the *HSP82* locus with twelve MS2 aptamer sequences. Upon RNA pulldown using immobilized streptavidin, we found that the levels of HSP mRNAs previously shown to coprecipitate with WT *HSP82* mRNA (e.g., *HSP104*, *HSP12*, *TMA10*, *SSA2*, and *SSA4* mRNAs) (*9*) were notably reduced in *hsp82^mut^* cells as compared to WT cells (Fig. 4F). These findings suggest that the conserved motif plays a crucial role in heat shock–induced HSP mRNA multiplexing and their ability to confer the HSR.

As a previous 3C experiment demonstrated that the *HSP82* gene interacts with other HSR genes, including *SSA2* and *HSP104* (*8*), we examined whether these interactions are affected by the *HSP82* mutation. We examined the coalescence of these genes in both the WT and mutant backgrounds using 3C and found no substantial differences in the level of interactions between the *HSP82* mutant and WT *HSP82* genes (fig. S5, B to G). Thus, mutations in the *HSP82* coding region that affect transperon formation do not appear to influence gene coalescence.

As our laboratory demonstrated that histone H4 interacts with transperon mRNAs and that histone H4 depletion inhibits transperon formation, mRNA colocalization, and transperon-related cell functions (e.g., mating and doubling time after heat shock) (*9*), we wanted to determine whether it acts prior to gene transcription (i.e., upon gene coalescence). To do so, we investigated whether histone H4 facilitates the coalescence of the heat shock genes upon heat shock. We used 3C to examine *HSP82* and *HSP104* or *SSA2* gene coalescence in strains bearing an auxin-induced degron fused to *HHF1* and lacking the *HHF2* H4 paralog. These cells remain fully viable for up to 4 hours upon auxin treatment but show a concomitant time-dependent loss of H4 histone family member 1 (Hhf1) protein (*9*). However, despite the critical role of histone H4 in driving the multiplexing of heat shock RNAs, the coalescence of HSP genes upon heat shock was unaffected (fig. S5, C and F). Thus, the necessity of histone H4 in transperon formation appears to be independent of heat shock–induced gene coalescence and likely relates to its ability to associate with transperon mRNAs (*9*).

### HSF1 drives multiplexing of HSP mRNAs

Hsf1 has two functional transcriptional activation domains located at the N- and C-terminal regions, known as the NTA and CTA, respectively. The *hsf1*Δ*NTA* (N-terminal deletion) mutant lacks the N-terminal activation domain (*27*), which is critical for HSR condensate formation (*6*). This mutant impairs Mediator and RNA Pol II recruitment, reducing intergenic interactions among the HSR genes (*6*, *28*). The *hsf1*Δ*CTA* (C-terminal deletion) mutant, missing its principal activation domain, also lacks the ability to effectively recruit mediator complex subunit 15 (Med15) and retinol-binding protein 1 (Rbp1), promote HSP gene transcription, and drive HSP gene interactions in heat-shocked cells (*6*, *28*).

**Fig. 5.**
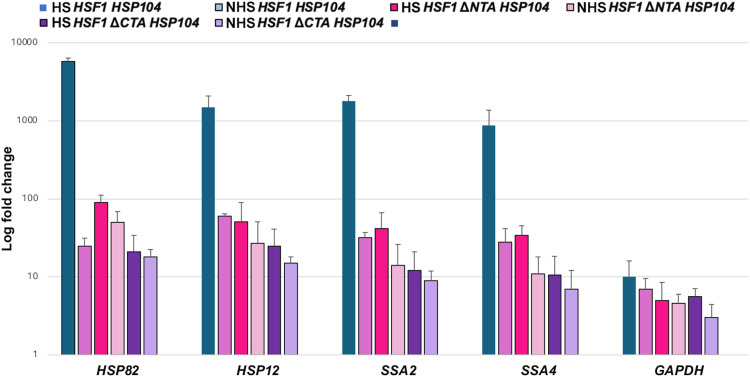
HSF1 drives multiplexing of heat shock mRNAs. WT *HSF1*, *HSF1*-Δ*NTA*, and *HSF1*-Δ*CTA* mutant cells expressing MS2 aptamer-tagged *HSP104* were either exposed to heat shock (10 min, 40°C; HS) or maintained at 30°C (NHS) prior to fixation and RaPID RNA pulldowns. RT-qPCR was performed using gene-specific oligonucleotides, and pulldown RNA levels were normalized to transcript levels in the TCL and are presented relative to the corresponding sample lacking the MS2 tag, which serves as a background control. Three biological replicates were performed. Error bars represent the SD.

Because HSP RNAs multiplex in both yeast and mammals, we hypothesized that HSF1 function drives transperon formation. We performed a RaPID pulldown experiment with tagged *HSP104* in WT, *hsf1*Δ*NTA*, and *hsf1*Δ*CTA* strain backgrounds in heat shock–treated cells. We found that HSP RNA multiplexing was essentially abolished in either of the mutants, being comparable to that observed in WT cells under non–heat shock conditions (Fig. 5). Therefore, we conclude that HSF1 function is critical for the formation of the heat shock RNA operon.

## DISCUSSION

**Fig. 6.**
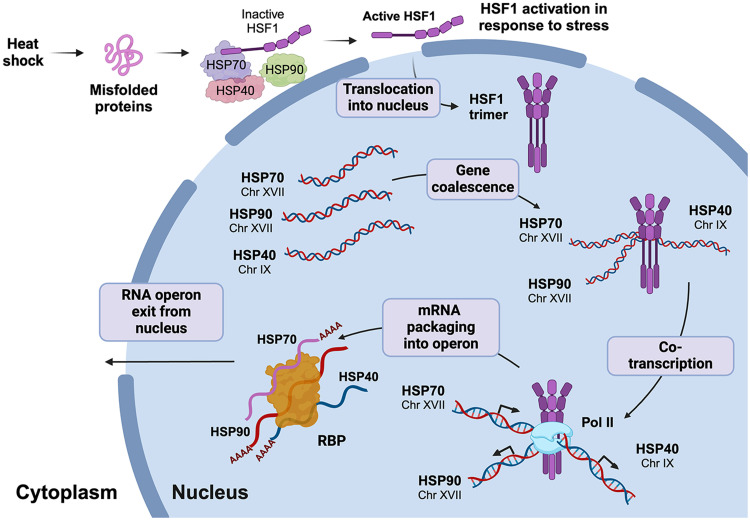
HSP mRNA multiplexing and operon formation are driven by HSF1-mediated HSP gene coalescence and cotranscription. In mammalian cells, inactive HSF1 monomers are retained in the cytoplasm in a complex with regulatory chaperones, such as HSP70, HSP90, and HSP40. In response to stress (e.g., heat shock), HSF1 dissociates from this inhibitory complex, trimerizes, and translocates to the nucleus. HSF1 then binds to HSEs of target HSP genes and, via liquid-liquid phase separation, mediates gene coalescence. HSF1-mediated gene coupling allows for interactions with RNA Pol II and the Mediator complex (latter not shown in schematic) and leads to coordinated transcription of the HSP genes and subsequent assembly of HSP mRNAs into an RNP complex, the HSR RNA operon (or transperon). Involvement of yet unknown RNA binding proteins (RBPs) is likely to play a central role in operon assembly. The RNA operon exits the nucleus and, upon translation, leads to the HSR. Upon translation of the HSPs and restoration of proteostasis, HSF1 is transported back to the cytoplasm and returned to its inactive state (not shown in schematic).

The present study explores the regulation of mammalian HSP gene cotranscription and mRNA multiplexing during the HSR, extending our earlier findings from yeast to mammalian systems. The proposed mechanism is summarized in Fig. 6, which visually illustrates the interactions and processes identified in this study. By demonstrating that HSP genes undergo HSF1-dependent interactions upon heat shock, subsequent mRNA assembly into RNA operons, and mRNA colocalization in the cytosol, our results highlight a complex evolutionarily conserved regulatory mechanism that governs stress-responsive gene expression.

We first confirmed that mammalian HSP genes engage in physical interactions, both between genes on the same chromosome and across different chromosomes, during heat shock. Using 3C analysis, we identified interactions between HSPA1A (HSP70) and HSP90AB1 (HSP90), as well as between HSPA1A and DNAJA1 (HSP40), both of which were disrupted upon HSF1 gene KO (Fig. 1). The HSF1-dependent coalescence of these genes is reminiscent of transcriptional condensates observed in both yeast and mammalian cells (*6*, *18*), suggesting that similar mechanisms may coordinate HSP gene expression across different genomic loci during stress. Consequently, we found that HSP mRNAs exhibit colocalization upon heat shock (Fig. 2). Using smFISH, we detected a substantial increase in colocalization between HSPA1A and HSP90AB1 mRNAs, as well as between the HSPA1A and HSPA8 mRNAs under heat shock conditions. Our findings support the hypothesis that akin to yeast transperons, mammalian cells also form heat shock–induced RNA-protein complexes that regulate the organization of mRNAs involved in stress responses. This idea is further supported by RNA pulldown assays, which demonstrated that mammalian HSP mRNAs multiplex to form an HSF1-dependent RNA operon under heat shock conditions. Specifically, HSPA1A mRNA coprecipitated other HSP mRNAs, such as HSP90AB1, DNAJA1, and HSPA8, with this association considerably enhanced after heat shock (Fig. 3B). The absence of HSF1 drastically reduced the formation of these complexes, underscoring the central role of HSF1 in orchestrating mRNA multiplexing during stress. These results align with previous work in yeast where HSF1 was shown to drive intra- and intergenic associations (*8*) and mRNA multiplexing during the HSR (*9*). Together, these findings demonstrate that heat shock triggers a coordinated cascade beginning with HSF1-dependent chromatin interactions among the HSP genes (as shown by 3C; Fig. 1), the assembly of multiplexed HSP mRNAs into stress-induced RNA operons (Fig. 3), and culminating in the colocalization of these mRNAs in the cytoplasm (Fig. 2). The absence of chromatin interactions under non–heat shock conditions (Fig. 1) highlights the inducible nature of this process, reinforcing the specificity and temporal ordering of chromatin coalescence, RNA operon formation, and mRNA colocalization.

In addition to demonstrating the conservation of this process, we also identified a conserved sequence motif within the coding regions of specific heat shock mRNAs that appears to facilitate the assembly of these ribonucleoproteins (RNPs) (Fig. 4). Through mutational analysis of *HSP82* in yeast, we showed that disruption of this motif not only altered RNA structure but also impaired the formation of multiplexed mRNAs, leading to a stress response phenotype that closely resembled that of an *hsp82*Δ deletion mutant. Mutation of this conserved motif did not affect heat shock–induced gene coalescence (fig. S5, C and F), suggesting that it likely plays a pivotal posttranscriptional role in mediating formation of the stress-responsive HSP mRNA operon and its function. Last, results obtained with deletion mutants of Hsf1 shown previously to affect transcriptional activation (*6*, *7*) demonstrated that enhanced transperon formation was abolished in heat shock–treated cells (Fig. 5), indicating that this transcription factor drives HSP mRNA expression and multiplexing upon activation.

Future directions for this work include determination of the role of histone H4 in transperon formation. In this work, we demonstrated that use of an inducible degron fused to the yeast *HHF1* histone H4 paralog did not abolish HSP gene coalescence upon induction in *hhf2*Δ cells (fig. S5, C and F), whereas our previous work showed that H4 depletion strongly inhibited transperon assembly (*9*). Thus, based on other earlier results showing that Hhf1 protein binds to individual transperon mRNAs, we suggest that the role of histone H4 is posttranscriptional, perhaps as RNA imprinting (*29*, *30*). However, the precise role of histone H4 in transperon assembly remains unclear and will require more study. Another direction for this work is to determine whether the HSP transperon mRNAs undergo colocalized cotranslation upon heat shock, as might be expected given their colocalization within both yeast and mammalian cells, as well as within intercellular connections formed between mammalian cells (i.e., TNTs), as shown in fig. S3D. The latter results suggest to us that fully formed transperons may transfer between cells and possibly confer cellular responsiveness, such as the HSR, to neighboring cells in vitro and perhaps in vivo.

Limitations to this current study include the necessity for further mutational analyses of conserved motifs found both in yeast as well as in mammalian cells to determine their potential roles in RNA assembly and transperon function. Correspondingly, the identification of proteins that might interact with these motifs using mass spectrometry might shed additional light on how RNA operons are assembled in eukaryotes. Previous Hi-C analysis of heat shock–treated mammalian cells did not reveal intrachromosomal or interchromosomal contacts (*31*), which might have been expected, given our 3C results. However, Hi-C may not provide clear evidence for these interactions since it measures interaction frequencies based on proximity across a cell population, potentially averaging out infrequent or transient DNA interactions. Also, incomplete digestions and DNA compaction can introduce background noise, and subsequent data filtering may reduce sensitivity and the detection of specific long-range interactions, a category to which long-range/spatially restricted intra- and interchromosomal interactions likely belong. In addition, while we found clear evidence of mRNA multiplexing and spatial coalescence of transcriptional loci under heat shock, the precise mechanisms underlying the spatial organization of transcription sites and the kinetics of RNP assembly remain to be resolved. Live-cell imaging approaches, combined with real-time RNA and protein tracking, may help capture the dynamics of this process with higher temporal and spatial resolution in future studies. Furthermore, although our data suggest the intercellular transfer of transperon particles (Fig. 5C), the physiological relevance and mechanism for this phenomenon in vivo requires further validation.

Together, our data suggest that mammalian cells use a highly coordinated mechanism to regulate heat shock gene expression through HSF1-mediated gene interactions, mRNA cotranscription, and the formation of RNP complexes (Fig. 6). This work expands our understanding of eukaryotic gene regulation under stress conditions, offering a model that draws parallels between prokaryotic operons and eukaryotic transperons. Moreover, the identification of a conserved motif that facilitates mRNA multiplexing provides a previously unknown layer of insight into how eukaryotic cells ensure efficient, synchronized responses to environmental stress. Demonstration of transperon formation first in yeast and now in higher eukaryotes is an important step in understanding combinatorial gene expression and its role in coordinating cellular processes. Aside from the HSR transperon, additional transperons were previously identified in yeast (*3*, *9*); thus, other mammalian cell pathways may be regulated in a similar manner.

Given the greater complexity of mammalian cells and growing need for medical intervention strategies, this work is important for our understanding of, and ability to manipulate, the HSR pathway that serves a critical function to relieve stress and refold denatured proteins in both normal and stressed cells. Specifically, the HSPs within the HSR are responsible for the folding, activation, and assembly of target proteins as ubiquitous molecular chaperones. HSP overexpression and inhibition can affect the regulation of apoptosis and therefore are targeted by a wide variety of therapies against pathological conditions including cancer, cellular aging, and senescence. It has not escaped our attention that interventions that reduce HSP transperon formation might also constitute an effective therapeutic strategy.

## MATERIALS AND METHODS

### Mammalian cell experiments

#### Cell lines and plasmids used in this study

Plasmids used in this study are listed in data file S1. Immortalized HSF^−/−^ (KO) MEFs (*32*) were a gift from R. Scherz-Shouval (Weizmann Institute of Science, Israel).

Human HSF1-GFP lentivirus vector was a gift from M. Vera Ugalde (McGill University, Canada). To create EF1α-HSF1-GFP (Addgene plasmid #227820), the human ubiquitin C (UbC) promoter driving the expression was replaced by the stronger EF1α promoter, which was PCR-amplified from pTwist-EF1α/Puro plasmid (Twist Bioscience), and subcloned using restriction enzymes Afe I and Not I.

EF1α-HSF1-GFP lentivirus particles were produced by transiently transfecting the expression plasmid with packaging plasmids, VSVG, RRE, and Rev (Addgene plasmids #12259, #12251, and #12253, respectively) into human embryonic kidney–293T cells using calcium phosphate and allowing the cells to grow for 72 hours. The virus-containing medium was harvested and concentrated with the Lenti-X concentrator (Clontech), per the manufacturer’s instructions. Viral particles were resuspended in complete Dulbecco’s modified Eagle’s medium, aliquoted, and stored at −80°C until infection.

For stable cell line generation, HSF1^−/−^ MEFs were seeded in 12-well plates and were exposed to the viral particles in serum-free medium containing polybrene (6 μg/ml; Sigma-Aldrich) for 2 hours with occasional shaking followed by addition of complete medium. Cells with high expression of the fluorescent reporter were selected by fluorescence-activated cell sorting (FACS) (BD Biosciences FACSAria III).

#### Transient transfection

Transient expression of the luciferase gene and the conserved HSE was carried out using the jetPRIME Transfection Reagent kit. The pGLuc and 6xHSE-Rluc plasmids (gifts from T. Czerny) (*33*) carrying six repeats of the conserved HSE were transiently cotransfected into WT MEFs or into HSF1^−/−^ mutant MEFs, before subjecting the cells to heat stress and the RNA pulldown procedure.

#### Chromatin conformation capture

The quantitative chromosome conformation capture method, Taq I–3C, (*34*) was used. MEF cells were first cultured at 37°C to 10^7^ cells in 10-cm plates and either maintained at that temperature or heat shocked for 1 hour at 42°C before being cross-linked with 1% formaldehyde for 10 min. Then, 125 mM glycine was added at room temperature for 5 min to stop the cross-linking. The cells were centrifuged at 1700*g* for 1 min and then subjected to lysis in formaldehyde (FA) lysis buffer [50 mM Hepes at pH 7.9, 140 mM NaCl, 1% Triton X-100, 0.1% sodium deoxycholate, 1 mM EDTA, and 1 mM phenylmethylsulfonyl fluoride (PMSF)] on ice for 20 min. Cell lysates were collected and centrifuged for 1 min at 1700*g*. The supernatant was transferred to a fresh Eppendorf tube and centrifuged for 10 min at 11,000*g* at 4°C. After centrifugation, a thin translucent layer of chromatin was observed on the top of the pellet of cell debris. The supernatant was discarded, and the pellet was resuspended in 1 ml of FA lysis buffer. The resuspended material was centrifuged at 13,000*g* for 10 min at 4°C, and the resulting pellet was resuspended in 500 μl of 1.2× Taq I restriction enzyme buffer. Next, 7.5 μl of 20% (w/v) SDS (final concentration of 0.3% SDS) was added, and the sample was incubated for 1 hour at 37°C with shaking (900 rpm). Then, 50 μl of 20% (v/v) Triton X-100 (final concentration = 1.8%) was added, and the sample was further incubated for 1 hour at 37°C with shaking (900 rpm). An undigested sample of genomic DNA (50 μl of aliquot) was removed and stored at −20°C to determine enzyme digestion efficiency. To the remaining sample, 200 U of Taq I (New England Biolabs) was added and incubated at 60°C for overnight. The next day, 150 μl of digested sample was heat-inactivated at 80°C for 20 min in the presence of added SDS (24 μl of 10% SDS; final concentration = 1.7%). To samples of digested material (174 μl each), 626 μl of 1.15× ligation buffer and 80 μl of 10% Triton X-100 (final concentration of 1%) were added and incubated at 37°C for 1 hour, while shaking gently. Proximity ligation in the sample was performed using 100 U of added T4 DNA ligase (New England Biolabs) at 16°C for 16 hours. The ligated samples were then digested with ribonuclease (RNase) [final concentration of 11 ng/μl; RNase A and RNAseT1 (28 U/μl); Thermo Fisher Scientific] at 37°C for 20 min. Proteinase-K (final concentration of 56 ng/μl; Sigma-Aldrich) digestion was performed at 65°C for 1 hour (note: final concentration of SDS = 0.4%). The 3C DNA template was extracted twice using an equal volume of phenol-chloroform (1:1 tris-EDTA-saturated phenol-chloroform, v/v), followed by extraction with an equal volume of phenol-chloroform–isoamyl alcohol (25:24:1, v/v) and by extraction with an equal volume of chloroform–isoamyl alcohol (24:1, v/v). The DNA was precipitated in the presence of 2 μl of glycogen (20 mg/ml), sodium acetate (0.3 M final concentration, pH 5.2), and 2.5 volumes of ethanol at room temperature overnight. The 3C DNA template obtained was stored at −20°C. DNA concentration was determined by absorption spectroscopy at 260 nm. Typically, 125 to 500 ng of the 3C DNA template was used in the PCR reactions. PCR products were eluted from agarose gels and sequenced for confirmation.

The frequency of chromatin interactions was determined using qPCR of ligation products generated during the 3C experiment. Primers specific to the ligation junctions were designed, and qPCR was performed using SYBR Green enzyme. For *P* value calculations, a Student’s *t* test (two-tailed, paired) was performed.

The interaction frequency was calculated using the following formula

$$\text{Relative Interaction Frequency}=2^{-\Delta Ct}$$

where **Δ*****Ct*** is defined as the difference between the ***Ct*** value of the ligation product (***Ct***_**sample**_) and the ***Ct*** value of a reference (***Ct***_**control**_). The reference was derived from a nonligation control.

To correct for potential differences in DNA digestion and ligation efficiency, a preligated control template containing the expected ligation junction was used to generate a standard curve for absolute quantification. PCR efficiency was also accounted for using the formula

$$\text{Corrected Interaction Frequency}={\left(1+E\right)}^{-\Delta Ct}$$

where *E* represents the primer efficiency obtained from the standard curve. The calculated interaction frequencies were normalized to an internal reference region. Experiments were performed in technical and biological replicates to ensure reproducibility and accuracy.

#### Single-molecule fluorescence in situ hybridization

smFISH in mammalian cells was carried out according to a previously described protocol (*21*, *22*, *35*). A set of Quasar 670–labeled FISH probes to detect HSPA1A or HSPA8 mRNA and Quasar 570–labeled FISH probes to HSP90AB1 mRNA were obtained from Stellaris. The sequences of the labeled nucleotides are provided in data file S1. MEF cells were maintained at 37°C or subjected to heat shock at 42°C for 1 hour, followed by a 1-hour recovery period at 37°C. For experiments examining transcription factor sites, the recovery step was omitted to capture immediate HSRs.

#### Widefield imaging

Images of smFISH experiments were captured using a Zeiss Axio Observer Z1 DuoLink dual camera imaging system equipped with an Illuminator HXP 120 V light source and Plan Apochromat 100 × 1.4 NA (numerical aperture) oil-immersion objective. Thirty 0.2-μm step *z*-stack images were taken for smFISH. For each individual experiment, between 100 and 150 cells were scored for mRNA and analyzed using the FISH-quant program (*20*) (https://bitbucket.org/muellerflorian/fish_quant). Colocalization between the two channels was calculated as a linear assignment problem solved with the Hungarian algorithm. We used MATLAB functions hungarianlinker^2^ and munkres^3^ developed by Mueller *et al.* (*20*) for this purpose. We also cross-checked colocalization (defined by overlap between the signals) manually for verification. For statistical analyses, at least three replicate experiments were carried out.

#### RNA pulldown using biotin-labeled DNA oligonucleotides

RNA pulldown using biotin-labeled oligonucleotides was performed following the protocol described by Torres *et al.* (*23*). Nine DNA oligonucleotide probes of 25 bases, which display strong affinity for the RNA of interest (e.g., HSPA1A), were designed as described and obtained as 3′-biotinylated oligonucleotides from Sigma-Aldrich. Next, 1 × 10^7^ cells maintained either at 37°C or shifted for 1 hour to 42°C were fixed using freshly prepared 0.1% paraformaldehyde solution in phosphate-buffered saline (PBS; 10 ml per culture plate) under agitation for 10 min at room temperature. Paraformaldehyde was quenched by adding 1/10 volume of 1.25 M glycine and incubated for 5 min at room temperature. Cells were rinsed two times (5 min each) with PBS. Cells were collected and centrifuged at 510*g* at 4°C for 5 min. The pellet was lysed for 15 min on ice in lysis buffer [50 mM tris-HCl (pH 7.0), 10 mM EDTA, 1% SDS supplemented with 200 U/ml of an RNase inhibitor solution, and a cocktail of protease inhibitors at 5 μl/ml] (cOmplete, EDTA-free protease inhibitor cocktail tablets; Sigma-Aldrich). Cells were centrifuged for 5 min at 12,000*g* at 4°C. Supernatants were hybridized with 100 pmol of each of the nine biotinylated oligonucleotides in two volumes of hybridization buffer [50 mM tris-HCl (pH 7.0), 750 mM NaCl, 1 mM EDTA, 1% SDS, and 10% formamide]. Prior to adding the probes, 20 μl from each sample were saved as input samples. Hybridization was allowed to proceed for 4 to 6 hours at 30°C. Following hybridization, 50 μl of Streptavidin-Sepharose high-performance beads (Sigma-Aldrich), supplemented with RNAase inhibitor solution (200 U/ml) and a cocktail of protease inhibitors (cOmplete, EDTA-free protease inhibitor cocktail tablets; Sigma-Aldrich) (5 μl/ml) were added to the samples and were incubated overnight under moderate agitation at room temperature. Beads were washed five times with 5 min of rotation each at room temperature with 900 μl of wash buffer (SDS 0.5% and SSC 2×). After the last wash, 95 μl of proteinase-K buffer [10 mM tris-HCl (pH 7.0), 100 mM NaCl, 1 mM EDTA, and 0.5% SDS] supplemented with 5 μl of proteinase-K (20 mg/ml) was added to the pulldown samples and 75 μl of supplemented proteinase-K buffer to the previously saved input samples. Samples were incubated at 50°C for 45 min then at 95°C for 10 min. Samples were placed on ice for 3 min, then total RNA was extracted using the NucleoSpin RNA Mini kit (Macherey Nagel) and RNA integrity checked using the Agilent TapeStation 2100, while the RNA concentration was measured using NanoDrop microvolume spectrophotometer and Qubit 2.0 broad-range assay. Reverse transcription using the UltraScript RT kit (PCR Biosystems) was followed by qPCR using specific primers (data file S1).

### Yeast cell experiments

#### Genetic manipulations and plasmids

Yeast strains created for this study were derived from WT BY4741 cells (see data file S1 for strain list and genotype). Standard lithium acetate-based protocols were used for the introduction of plasmids and PCR products into yeast. For the RaPID RNA pulldown experiment (*25*), cells were grown at 30°C to mid-log phase [optical density at 600 nm (OD_600_) = ~1] in a standard rich growth medium containing 2% glucose [yeast extract, peptone, and dextrose (YPD)] or in a synthetic medium containing 2% glucose [e.g., synthetic complete (SC)] and selective dropout medium lacking amino acid. Induction of the methionine starvation-inducible plasmid [pUG36-MS2-CP-GFP(x3)] was performed by transferring cells to mid-log phase to a synthetic medium lacking methionine and subsequent growth of the cells for 1 hour with shaking at 30°C. Any genomic manipulation used for creating the strains for the RaPID pulldown experiment was done following the protocol detailed in (*36*). Cells were grown at 30°C or heat shocked (in preheated 50°C medium) at 40°C for 10 min; see below for the RaPID protocol used.

For growth tests on plates, 2 × 10^7^ yeast cells were grown to mid-log phase, normalized for absorbance at OD_600_, serially diluted 1:10, plated by drops onto solid medium, and grown at the indicated temperatures for 48 hours before photodocumentation. Three biological replicas were performed for each growth test. For the quantitative growth assay, cells were grown in 250-ml Erlenmeyer flasks in 50 ml of YPD medium at the indicated temperatures, with or without 1 M KCl at 30°C, for up to 26 hours with constant shaking. Absorbance at OD_600_ was measured at specified time points to monitor growth.

Chromosomal integration of the four different point mutations into the conserved HSP motif sequence of *HSP82* was executed using standard techniques, based on homologous recombination. We first obtained a fragment containing the four point mutations using WT genomic DNA as template for two separate PCR reactions. One reaction used primer (5′-ATTTCTTCCCGCTGTATTAGAGTTC) together with primer (5′-ACAAGTCGAA**C**GGTGCTCTCTT**G**GGAATGAATAA**A**ATAGCTCTAAATTCCAATTG**C**CCTTCAACGGAG), and a second reaction used primer (5′-GCAGATGCCCTATTTACATACTTAT) together with primer (5′-CTCCGTTGAAGG**G**CAATTGGAATTTAGAGCTAT**T**TTATTCATTCC**C**AAGAGAGCACC**G**TTCGACTTGT). The underlined and bolded nucleotide bases indicate the site we inserted the silent point mutations. For the PCR reaction, 34 cycles of PCR were performed with the following temperature profile: 94°C, 1 min; 54°C, 1 min; and 72°C, 2 min. The resulting two PCR fragments were stitched together using 1 μl of PCR product from each PCR reaction as template and primers #1 and #4. PCR amplification was achieved by 34 cycles of PCR with the following temperature profile: 94°C, 1 min; 48°C, 1 min; and 72°C, 2 min. The PCR product was transformed into the *hsp82*Δ::*URA3* strain and plated onto 5-fluoroorotic acid (5-FOA) plates for selection against *URA3* cells to yield *hsp82^mut^*::*ura3* cells. Genomic DNA was isolated from cells that grew in the presence of 5-FOA and were further subjected to DNA sequence analysis to ensure the presence of the point mutations at the correct locus. The *hsp82*Δ::*URA3* strain was created by amplifying the *URA3* gene from pCG plasmid by PCR using the following primers: forward, 5′-GTCCTATAAACAAAAGCACAAACAAACACGCAAAGATATG*CACAGGAAACAGCTATGACC*, and reverse, 5′-TTTTGTTTATAACCTATTCAAGGCCATGATGTTCTACCTA*GTTGTAAAACGACGGCCAGT*. The PCR product was transformed into BY4741 cells and plated onto minimal medium lacking uracil. Integration of the *URA3* gene was confirmed by PCR with the following primers: forward, 5′-ATAACTTAGCTTGCGTGTTGCGT, and reverse, 5′-GTACGAACATCCAATGAAGCACACA.

#### RaPID procedure for the precipitation of RNP complexes

The pulldown of MS2 aptamer-tagged mRNAs and detection for bound RNAs and proteins were performed using the RaPID procedure, essentially as described in (*25*, *37*). Yeast strains bearing endogenously expressed genes tagged with the MS2 aptamer created based on the protocol detailed in (*36*) were grown in a volume of 400 ml to mid-log phase at 26°C with constant shaking to an OD_600_ = ~0.8. Cells were transferred to preheated medium and then subjected to heat shock at 50°C for 10 min. Cells were centrifuged in a Sorvall SLA-3000 rotor at 1100*g* for 5 min, resuspended in 200 ml of complete synthetic medium lacking methionine to induce the expression of the MS2-CP-GFP-SBP protein, and grown for an additional 45 min. The cells were collected by centrifugation as described above, washed with PBS buffer (lacking Ca^++^ and Mg^++^), and transferred into a 50-ml tube and pelleted as above. Proteins were cross-linked by the addition of 8 ml of PBS containing 0.01% formaldehyde and incubated at room temperature for 10 min with slow shaking. The cross-linking reaction was terminated by adding 1 M glycine buffer (pH = 8.0) to a final concentration of 0.125 M with additional shaking for 2 min. The cells were then washed once with ice-cold PBS buffer, and the pellet was flash frozen in liquid nitrogen and stored at −80°C.

For cell lysis and RNA pulldown, cell pellets were thawed upon the addition of ice-cold lysis buffer [20 mM tris-HCl at pH 7.5, 150 mM NaCl, 1.8 mM MgCl_2_, and 0.5% NP-40 supplemented with aprotinin (10 mg/ml), 1 mM PMSF, pepstatin A (10 mg/ml), leupeptin (10 mg/ml), 1 mM dithiothreitol (DTT), and RNAsin (80 U/ml; Promega)] at 1 ml per 100 OD_600_ unit, and 0.5-ml aliquots were then transferred to separate microcentrifuge tubes containing an equal volume of 0.5-mm glass beads, and vortexed in a Vortex Genie Cell Disruptor (Scientific Instruments) shaker at maximum speed for 10 min at 4°C. Glass beads and unbroken cells were sedimented at 4°C by centrifugation at 1700*g* for 1 min, and the supernatant was transferred to fresh microcentrifuge tubes and centrifuged at 11,000*g* at 4°C for 10 min. The total cell lysate (TCL) was then transferred to a fresh tube, and protein concentration was determined using the Micro BCA protein determination kit (Pierce). Protein (10 mg of TCL) was taken per pulldown reaction. To block endogenous biotinylated moieties, protein aliquots were incubated with 10 μg of free avidin (Sigma-Aldrich) per 1 mg of input protein for 1 hour at 4°C with constant rotation. In parallel, streptavidin-conjugated beads (Streptavidin-Sepharose high-performance beads, GE Healthcare) were aliquoted to microcentrifuge tubes according to 5 μl of slurry per 1 mg of protein (not >30 μl overall), washed twice with 1 ml of PBS, once with 1 ml of lysis buffer, and blocked with a 1:1 mixture of 1 ml of lysis buffer containing yeast tRNA (Sigma-Aldrich; 0.1 mg/100 ml of beads) and 1 ml of 4% bovine serum albumin in PBS at 4°C for 1 hour with constant rotation. Following blocking, beads were washed twice in 1 ml of lysis buffer. Pulldown was performed by adding the indicated amount of avidin-blocked TCL to the beads, followed by incubation at 4°C for 2 to 15 hours with constant rotation. Yeast tRNA was added to the pulldown reaction (0.1 mg per tube) to reduce nonspecific interactions. We used standard 1.7-ml microcentrifuge tubes when working with small volumes of TCL or 15-ml sterile polypropylene centrifuge tubes with larger volumes. Following pulldown, the beads were centrifuged at 660*g* at 4°C for 2 min, the supernatant was then removed, and the beads were washed three times with lysis buffer (e.g., 1-ml volume washes for small tubes and 2 ml for large tubes) and twice with wash buffer [20 mM tris (pH 7.5), 300 mM NaCl, and 0.5% Triton X-100], all performed at 4°C (each step lasting 10 min with rotation). The beads were then equilibrated by a final wash in 1 to 2 ml of cold PBS, pelleted by centrifugation as above, and excess buffer was aspirated. For elution of the cross-linked RNP complexes from the beads, 150 ml of PBS containing 6 mM free biotin (Sigma-Aldrich) was added to the beads, followed by 1 hour of incubation at 4°C with rotation. After centrifugation at 660*g* for 2 min, the eluate was transferred into a fresh microcentrifuge tube, recentrifuged, and transferred into another tube to assure that no beads were carried over. To reverse the cross-link, the eluate was incubated at 70°C for 1 to 2 hours with an equal volume of 2× cross-link reversal buffer [100 mM tris (pH 7.0), 10 mM EDTA, 20 mM DTT, and 2% SDS] for RNA analysis or with an appropriate volume of 5× protein sample buffer (5×: 0.4 M tris at pH 6.8, 50% glycerol, 10% SDS, 0.5 M DTT, and 0.25% bromophenol blue) for protein analysis using SDS–polyacrylamide gel electrophoresis. For RaPID pulldowns and RT-PCR analysis, three biological replicas were performed each, and the average ± SD is given. Primers used for qPCR reactions are listed in data file S1.
