# Supplementary material for: Conservation of mRNA operon formation in control of the heat shock response in mammalian cells
Source: Sci Adv. 2025 Dec 5;11(49):eadu0315. doi: 10.1126/sciadv.adu0315 (PMC12680049; doi:10.1126/sciadv.adu0315)
Supplement: Supplementary file 1 — Figs. S1 to S5 Legend for data file S1 [file sciadv.adu0315_sm.pdf]

Science Advances
AAAS

Supplementary Materials for

Conservation of mRNA operon formation in control of the heat shock response in mammalian cells

Emese Pataki and Jeffrey E. Gerst

Corresponding author: Jeffrey E. Gerst, jeffrey.gerst@weizmann.ac.il

Sci. Adv. **11**, eadu0315 (2025)
DOI: 10.1126/sciadv.adu0315

The PDF file includes:

Figs. S1 to S5
Legend for data file S1

Other Supplementary Material for this manuscript includes the following:

Data file S1

Supplementary Figures

A.

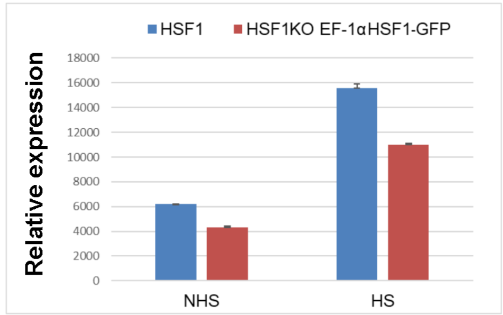

B.

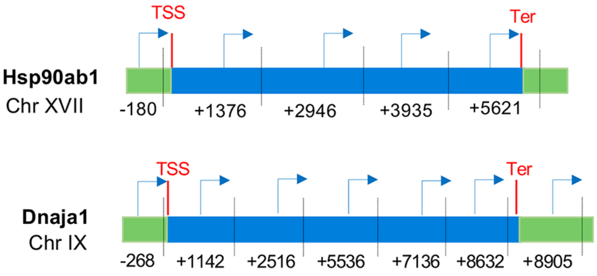

DNAJA1 \ HSP90AB1	1	2	3	4	5
1	-	-	-	-	-
2	-	-	-	-	-
3	-	-	-	-	-
4	-	-	-	-	-
5	-	-	-	-	-
6	-	-	-	-	-
7	-	-	-	-	-

Fig. S1.
(A) Expression levels of endogenous HSF1 and exogenous EF-1α-HSF1-GFP in HSF1 knockout MEFs. Mouse embryonic fibroblasts (MEFs) were either maintained at 37°C or exposed to heat shock at 42°C for 1 hour. Total RNA was then extracted, and mRNA expression levels were quantified by qRT-PCR and normalized to those of GAPDH. Three independent biological replicates were performed. Error bars represent the standard deviation. **(B) Lack of interaction between the HSP90AB1 and DNAJA1 genes upon heat shock in MEFs, as determined by the Chromatin conformation capture (3C) technique.** Top panel: Graphic illustration of the HSP90AB1 and DNAJA1 genes, and the oligonucleotides used for their

amplification from 3C DNA samples. Coordinates correspond to TaqI sites (shown as vertical dashed bars); site numbering is relative to ATG (+1). Forward primers used for 3C analysis were sense-strand identical (arrows) and positioned proximal to TaqI sites as indicated. Primers are numbered to distinguish the pairs used in the PCR reactions shown in matrix. 5'UTRs, ORFs, and 3'UTRs are color-coded, as indicated. Transcription start sites (TSS) and termination sites (Ter) are indicated. Bottom panel: A matrix summarizing the intergenic associations of represented genes as determined by 3C-PCR. Primer pairs corresponding to the different genes listed in were used in PCR reactions. '–' indicates no amplification. GAPDH primers were used as a control for the PCR reaction (not shown).

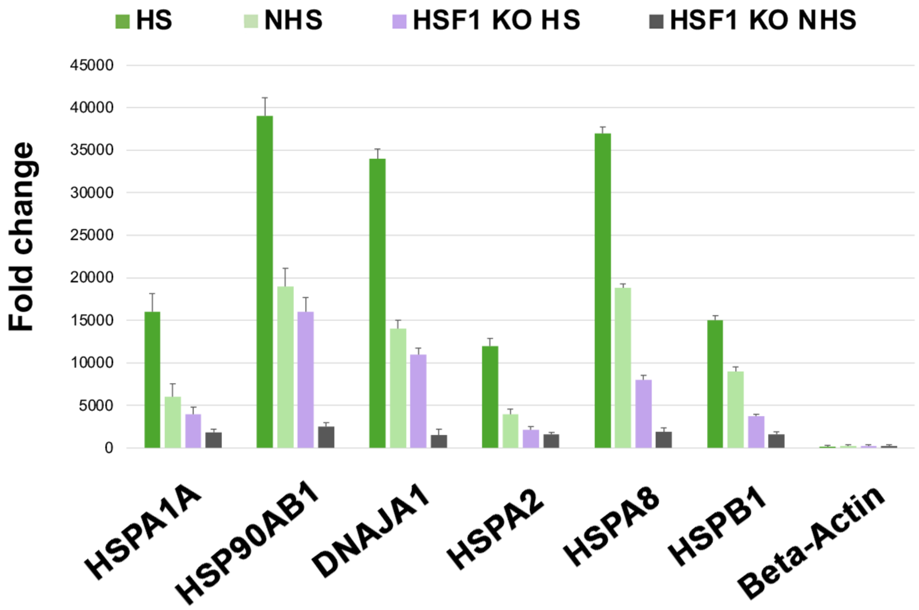

Fig. S2.

Expression levels of HSP genes in heat-shocked and non-heat-shocked cells. Both wild-type (WT) and HSF1 knockout (HSF1$^{-/-}$) mouse embryonic fibroblasts (MEFs) were either maintained at 37°C or subjected to heat shock at 42°C for 1 hour. Total RNA was extracted, and mRNA expression levels were quantified by qRT-PCR using gene-specific oligonucleotides with normalization to those of GAPDH. Three independent biological replicates were performed, and error bars represent the standard deviation. For pulldown samples, mRNA levels were normalized to those in the total cell extract.

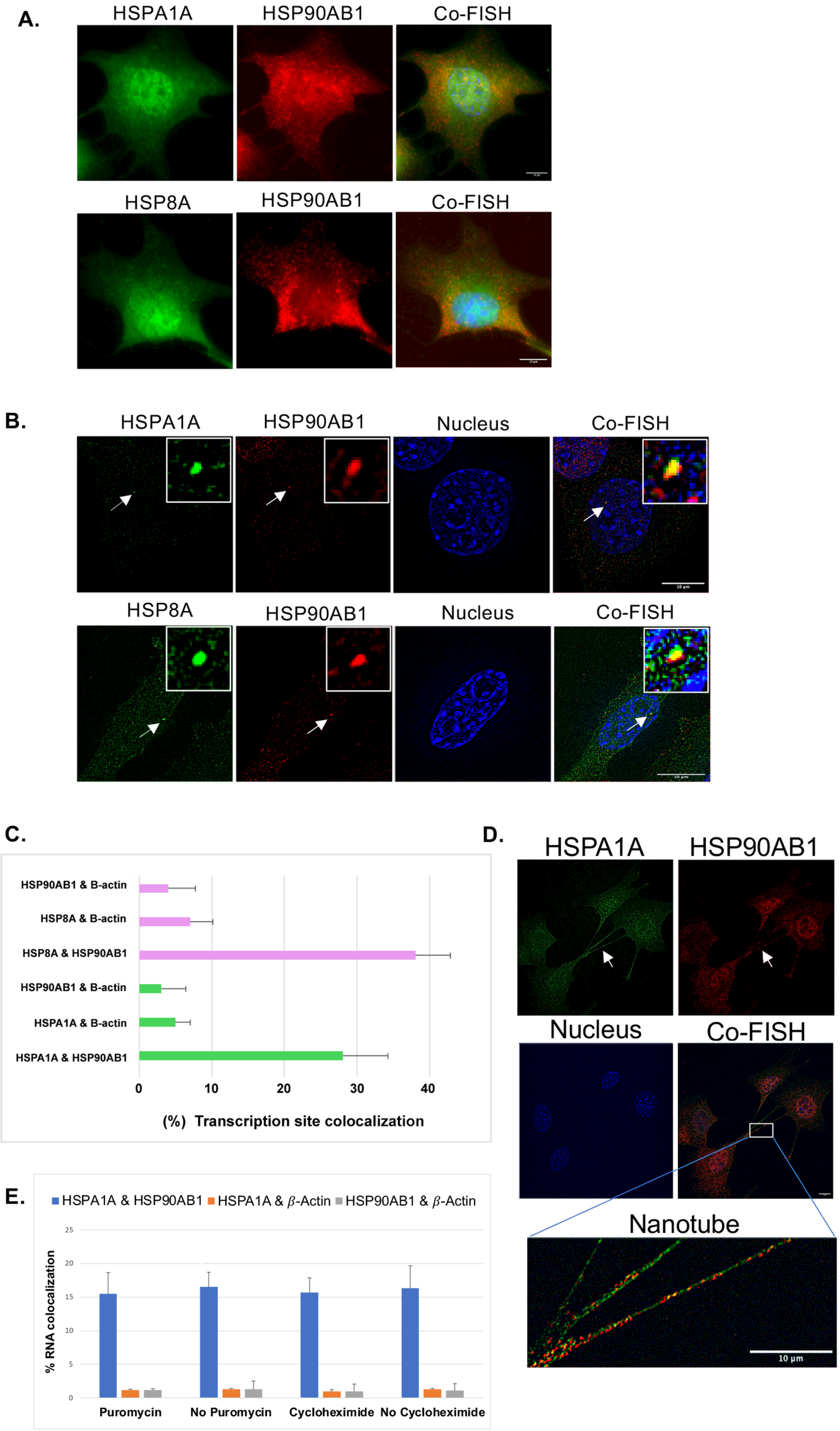

A.
HSPA1A
HSP90AB1
Co-FISH
HSP8A
HSP90AB1
Co-FISH
B.
HSPA1A
HSP90AB1
Nucleus
Co-FISH
HSP8A
HSP90AB1
Nucleus
Co-FISH
C.
HSP90AB1 & B-actin
HSP8A & B-actin
HSP8A & HSP90AB1
HSP90AB1 & B-actin
HSPA1A & B-actin
HSPA1A & HSP90AB1
0
10
20
30
40
(%) Transcription site colocalization
D.
HSPA1A
HSP90AB1
Nucleus
Co-FISH
Nanotube
10 µm
E.
HSPA1A & HSP90AB1
HSPA1A & β-Actin
HSP90AB1 & β-Actin
% RNA colocalization
25
20
15
10
5
0
Puromycin
No Puromycin
Cycloheximide
No Cycloheximide

Fig. S3.

HSP mRNAs and transcription sites co-localize upon heat shock. (A) Representative single-molecule fluorescence *in situ* hybridization (smFISH) images of MEFs under non-heat shock (NHS) conditions. Cells were labeled with sequence-specific FISH probes complementary to HSPA1A, HSP90AB1, and HSPA8 mRNAs, followed by DAPI staining of the nucleus (blue). HSPA1A and HSPA8 were labeled with Quasar® 670 (Cy5, green), while HSP90AB1 was labeled with Quasar® 570 (Cy3, red). Merged signals from all three probes are labeled "Co-FISH". The size bar indicates 10μm.

(B) Representative smFISH images of MEFs after heat shock, demonstrating the co-localization of HSP90AB1 and the HSPA1A and HSPA8 mRNAs colocalized to transcription sites within the DAPI-labeled nucleus. The size bar indicates 10μm.

(C) Histogram of the data obtained from 3 biological replicates.

(D) Representative smFISH images of MEFs after heat shock, demonstrating the co-localization of HSPA1A and HSP90AB1 mRNAs within a nanotube, as indicated by the arrow.

(E) A histogram summarizes data obtained from three biological replicates of the smFISH experiment. MEFs subjected to heat shock, treated with either puromycin (10 μg/mL) or cycloheximide (100 μg/mL) for 30min prior to fixation. Cells were processed for smFISH labeling using sequence- specific FISH probes complementary to HSPA1A, HSP90AB1, prior to labeling with DAPI. The mRNA was quantified using FISH-Quant.

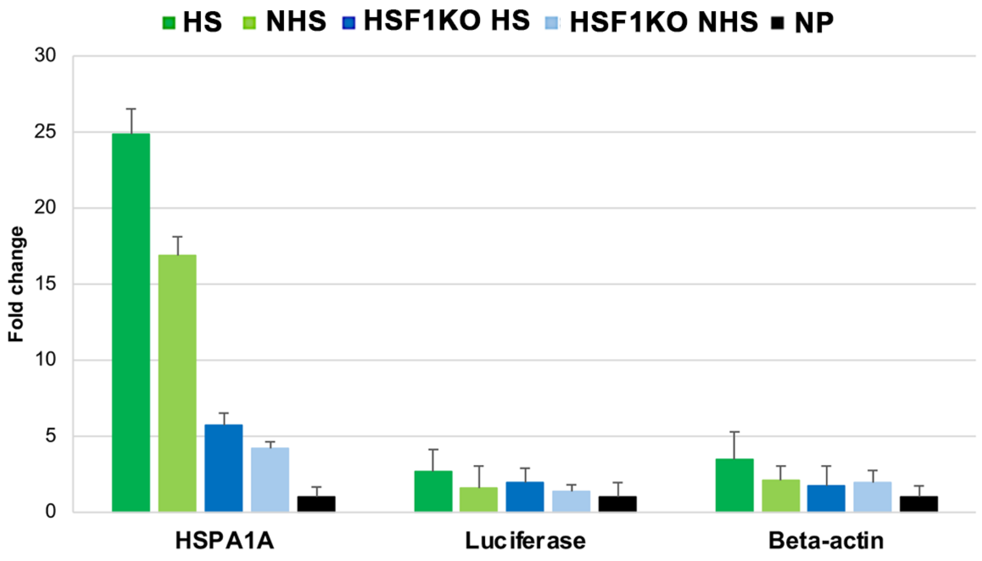

Fig. S4.

HSF1 and the HSP promoter containing a HSE drive HSP RNA multiplexing. The pGLuc luciferase plasmid lacking the HSE was transiently expressed in WT and HSF1$^{-/-}$ KO MEF cells and either subjected to heat shock at 42°C for 1hr and recovery at 37°C for 1hr or maintained at 37 °C prior to fixation and the RNA pulldown procedure. qRT-PCR was performed using specific oligonucleotides and expression levels were quantified following normalization with those of the total cell lysate relative to the "no probe "control (negative control). Three biological replicates were performed, and error bars indicate standard deviation.

.

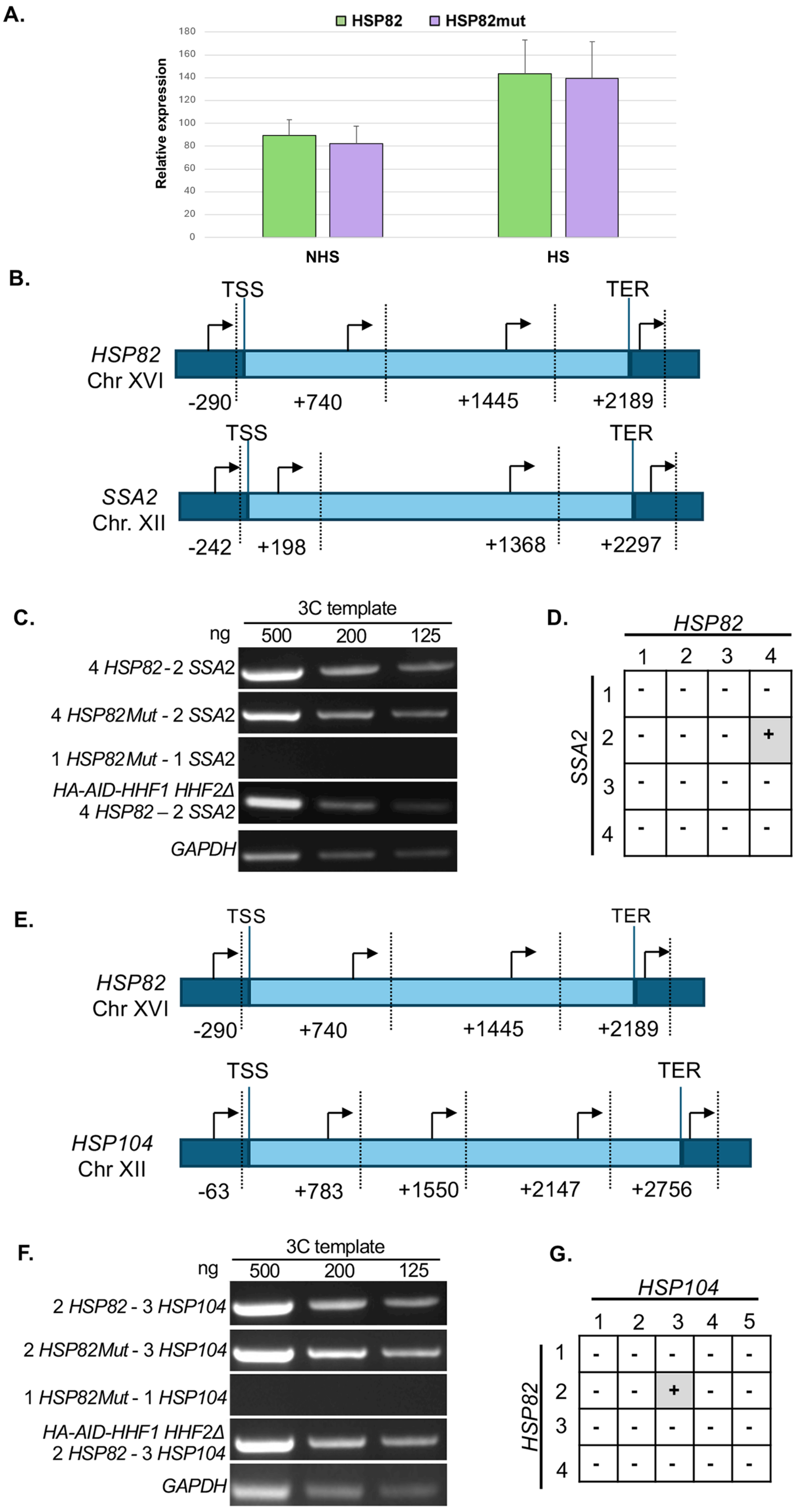
A.
HSP82
HSP82mut
Relative expression
180
160
140
120
100
80
60
40
20
0
NHS
HS
B.
TSS
TER
HSP82
Chr XVI
-290
+740
+1445
+2189
TSS
TER
SSA2
Chr. XII
-242
+198
+1368
+2297
C.
3C template
ng
500
200
125
4 HSP82 - 2 SSA2
4 HSP82Mut - 2 SSA2
1 HSP82Mut - 1 SSA2
HA-AID-HHF1 HHF2Δ
4 HSP82 – 2 SSA2
GAPDH
D.
HSP82
SSA2
1
2
3
4
+
E.
TSS
TER
HSP82
Chr XVI
-290
+740
+1445
+2189
TSS
TER
HSP104
Chr XII
-63
+783
+1550
+2147
+2756
F.
3C template
ng
500
200
125
2 HSP82 - 3 HSP104
2 HSP82Mut - 3 HSP104
1 HSP82Mut - 1 HSP104
HA-AID-HHF1 HHF2Δ
2 HSP82 - 3 HSP104
GAPDH
G.
HSP104
HSP82
1
2
3
4
5
+

Fig. S5.

Heat shock-induced HSP gene coalescence is unaffected by mutations in HSP82 or in histone H4. (A) Expression levels of *HSP82* WT and *HSP82* motif mutant (HSP82mut) transcripts were assessed by qRT-PCR under heat shock (HS) and non-heat shock (NHS) conditions. Transcript levels were normalized to *GAPDH* and are presented as relative expression values. Data represent the mean of three biological replicates, and error bars indicate the standard deviation. (B-G) 3C assays were performed to examine interactions between the *HSP82* gene with the *HSP104* or *SSA2* genes. These were examined both in WT yeast or yeast bearing an auxin-induced degron fused to *HHF1* and lacking the *HHF2* gene (histone H4 paralog), as well as a strain with point mutations in *HSP82* (*HSP82Mut*). (B, E) Graphic illustration of the *HSP82* and *SSA2* or *HSP82* and *HSP104* genes, and the oligonucleotides used for their amplification from 3C DNA samples. Coordinates correspond to TaqI sites (shown as vertical dashed bars); site numbering is relative to ATG (+1). Forward primers used for 3C analysis were sense-strand identical (arrows) and positioned proximal to TaqI sites as indicated. Primers are numbered to distinguish the pairs used in the PCR reactions shown in (C, F). 5'UTRs, ORFs, and 3'UTRs are color-coded, as indicated. Transcription start sites (TSS) and termination sites (Ter) indicated. (D, G) Matrices summarizing the intergenic associations of represented genes as determined by 3C-PCR. Primer pairs corresponding to the different genes listed in (B, E) were used in PCR reactions. '+' indicates PCR amplification and the interaction between genes. '–' indicates no amplification. (D, G) PCR products derived reactions using the indicated primer pairs (to the genes shown in B, E) and 3C-processed DNA were electrophoresed on agarose gels (1%) and visualized by ethidium bromide staining. Lanes represent the 3C-PCR output using the indicated concentrations of DNA template (ng DNA). *GAPDH* primers were used as a control for PCR reactions.

Supplementary Data File S1. (see separate file)
